# Supplementary material for: HIVprotI: an integrated web based platform for prediction and design of HIV proteins inhibitors
Source: J Cheminform. 2018 Mar 9;10:12. doi: 10.1186/s13321-018-0266-y (PMC5845081; doi:10.1186/s13321-018-0266-y)
Supplement: Supplementary file 2 — Additional file 2. Source code of HIVProtI web server. [file 13321_2018_266_MOESM2_ESM.zip › HIVprotI_Source-code/hivprotI/weka-3-6-6/doc/weka/gui/beans/Loader.html]

Loader


---


|  |  |  |
| --- | --- | --- |
| **Overview** \| **Package** \| **Class** \| **Tree** \| **Deprecated** \| **Index** \| **Help** \| **Weka's home** | |  |
| **PREV CLASS**   **NEXT CLASS** | **FRAMES**    **NO FRAMES**     **All Classes** |  |
| SUMMARY: NESTED \| FIELD \| CONSTR \| METHOD | DETAIL: FIELD \| CONSTR \| METHOD |  |


---


## weka.gui.beans Class Loader

```
java.lang.Object
  java.awt.Component
      java.awt.Container
          javax.swing.JComponent
              javax.swing.JPanel
                  weka.gui.beans.AbstractDataSource
                      weka.gui.beans.Loader
```

**All Implemented Interfaces:**: java.awt.image.ImageObserver, java.awt.MenuContainer, java.beans.beancontext.BeanContextChild, java.io.Serializable, javax.accessibility.Accessible, EnvironmentHandler, BeanCommon, DataSource, EventConstraints, Startable, StructureProducer, Visible, WekaWrapper

---

``` public class Loader extends AbstractDataSource implements Startable, WekaWrapper, EventConstraints, BeanCommon, EnvironmentHandler, StructureProducer ```

Loads data sets using weka.core.converter classes

**Since:**
:   1.0

**Version:**
:   $Revision: 7458 $

**Author:**
:   Mark Hall

**See Also:**: `AbstractDataSource`, `UserRequestAcceptor`, Serialized Form

---

**Nested Class Summary**

**Nested classes/interfaces inherited from class javax.swing.JComponent**

`javax.swing.JComponent.AccessibleJComponent`

**Nested classes/interfaces inherited from class java.awt.Component**

`java.awt.Component.BaselineResizeBehavior`


**Field Summary**

**Fields inherited from class javax.swing.JComponent**

`TOOL_TIP_TEXT_KEY, UNDEFINED_CONDITION, WHEN_ANCESTOR_OF_FOCUSED_COMPONENT, WHEN_FOCUSED, WHEN_IN_FOCUSED_WINDOW`

**Fields inherited from class java.awt.Component**

`BOTTOM_ALIGNMENT, CENTER_ALIGNMENT, LEFT_ALIGNMENT, RIGHT_ALIGNMENT, TOP_ALIGNMENT`

**Fields inherited from interface java.awt.image.ImageObserver**

`ABORT, ALLBITS, ERROR, FRAMEBITS, HEIGHT, PROPERTIES, SOMEBITS, WIDTH`


**Constructor Summary**

`Loader()`


| **Method Summary** | |
| --- | --- |
| `void` | `addDataSourceListener(DataSourceListener dsl)`             Add a listener |
| `void` | `addInstanceListener(InstanceListener dsl)`             Add an instance listener |
| `boolean` | `connectionAllowed(java.beans.EventSetDescriptor esd)`             Returns true if, at this time, the object will accept a connection via the supplied EventSetDescriptor. |
| `boolean` | `connectionAllowed(java.lang.String eventName)`             Returns true if, at this time, the object will accept a connection via the named event |
| `void` | `connectionNotification(java.lang.String eventName, java.lang.Object source)`             Notify this object that it has been registered as a listener with a source for receiving events described by the named event This object is responsible for recording this fact. |
| `void` | `disconnectionNotification(java.lang.String eventName, java.lang.Object source)`             Notify this object that it has been deregistered as a listener with a source for named event. |
| `boolean` | `eventGeneratable(java.lang.String eventName)`             Returns true if the named event can be generated at this time |
| `java.lang.String` | `getCustomName()`             Get the custom (descriptive) name for this bean (if one has been set) |
| `Loader` | `getLoader()`             Get the loader |
| `java.lang.String` | `getStartMessage()`             Gets a string that describes the start action. |
| `Instances` | `getStructure(java.lang.String eventName)`             Get the structure of the output encapsulated in the named event. |
| `java.lang.Object` | `getWrappedAlgorithm()`             Get the loader |
| `java.lang.String` | `globalInfo()`             Global info (if it exists) for the wrapped loader |
| `boolean` | `isBusy()`             Returns true if. |
| `static void` | `main(java.lang.String[] args)` |
| `void` | `removeDataSourceListener(DataSourceListener dsl)`             Remove a listener |
| `void` | `removeInstanceListener(InstanceListener dsl)`             Remove an instance listener |
| `void` | `setBeanContext(java.beans.beancontext.BeanContext bc)`             Set a bean context for this bean |
| `void` | `setCustomName(java.lang.String name)`             Set a custom (descriptive) name for this bean |
| `void` | `setDB(boolean flag)` |
| `void` | `setEnvironment(Environment env)`             Set environment variables to use. |
| `void` | `setLoader(Loader loader)`             Set the loader to use |
| `void` | `setLog(Logger logger)`             Set a logger |
| `void` | `setWrappedAlgorithm(java.lang.Object algorithm)`             Set the loader |
| `void` | `start()`             Start loading |
| `void` | `startLoading()`             Start loading data |
| `void` | `stop()`             Stop any loading action. |

**Methods inherited from class weka.gui.beans.AbstractDataSource**

`addPropertyChangeListener, addVetoableChangeListener, getBeanContext, getVisual, removePropertyChangeListener, removeVetoableChangeListener, setVisual, useDefaultVisual`

**Methods inherited from class javax.swing.JPanel**

`getAccessibleContext, getUI, getUIClassID, setUI, updateUI`

**Methods inherited from class javax.swing.JComponent**

`addAncestorListener, addNotify, addVetoableChangeListener, computeVisibleRect, contains, createToolTip, disable, enable, firePropertyChange, firePropertyChange, firePropertyChange, getActionForKeyStroke, getActionMap, getAlignmentX, getAlignmentY, getAncestorListeners, getAutoscrolls, getBaseline, getBaselineResizeBehavior, getBorder, getBounds, getClientProperty, getComponentPopupMenu, getConditionForKeyStroke, getDebugGraphicsOptions, getDefaultLocale, getFontMetrics, getGraphics, getHeight, getInheritsPopupMenu, getInputMap, getInputMap, getInputVerifier, getInsets, getInsets, getListeners, getLocation, getMaximumSize, getMinimumSize, getNextFocusableComponent, getPopupLocation, getPreferredSize, getRegisteredKeyStrokes, getRootPane, getSize, getToolTipLocation, getToolTipText, getToolTipText, getTopLevelAncestor, getTransferHandler, getVerifyInputWhenFocusTarget, getVetoableChangeListeners, getVisibleRect, getWidth, getX, getY, grabFocus, isDoubleBuffered, isLightweightComponent, isManagingFocus, isOpaque, isOptimizedDrawingEnabled, isPaintingForPrint, isPaintingTile, isRequestFocusEnabled, isValidateRoot, paint, paintImmediately, paintImmediately, print, printAll, putClientProperty, registerKeyboardAction, registerKeyboardAction, removeAncestorListener, removeNotify, removeVetoableChangeListener, repaint, repaint, requestDefaultFocus, requestFocus, requestFocus, requestFocusInWindow, resetKeyboardActions, reshape, revalidate, scrollRectToVisible, setActionMap, setAlignmentX, setAlignmentY, setAutoscrolls, setBackground, setBorder, setComponentPopupMenu, setDebugGraphicsOptions, setDefaultLocale, setDoubleBuffered, setEnabled, setFocusTraversalKeys, setFont, setForeground, setInheritsPopupMenu, setInputMap, setInputVerifier, setMaximumSize, setMinimumSize, setNextFocusableComponent, setOpaque, setPreferredSize, setRequestFocusEnabled, setToolTipText, setTransferHandler, setVerifyInputWhenFocusTarget, setVisible, unregisterKeyboardAction, update`

**Methods inherited from class java.awt.Container**

`add, add, add, add, add, addContainerListener, addPropertyChangeListener, applyComponentOrientation, areFocusTraversalKeysSet, countComponents, deliverEvent, doLayout, findComponentAt, findComponentAt, getComponent, getComponentAt, getComponentAt, getComponentCount, getComponents, getComponentZOrder, getContainerListeners, getFocusTraversalKeys, getFocusTraversalPolicy, getLayout, getMousePosition, insets, invalidate, isAncestorOf, isFocusCycleRoot, isFocusCycleRoot, isFocusTraversalPolicyProvider, isFocusTraversalPolicySet, layout, list, list, locate, minimumSize, paintComponents, preferredSize, printComponents, remove, remove, removeAll, removeContainerListener, setComponentZOrder, setFocusCycleRoot, setFocusTraversalPolicy, setFocusTraversalPolicyProvider, setLayout, transferFocusBackward, transferFocusDownCycle, validate`

**Methods inherited from class java.awt.Component**

`action, add, addComponentListener, addFocusListener, addHierarchyBoundsListener, addHierarchyListener, addInputMethodListener, addKeyListener, addMouseListener, addMouseMotionListener, addMouseWheelListener, bounds, checkImage, checkImage, contains, createImage, createImage, createVolatileImage, createVolatileImage, dispatchEvent, enable, enableInputMethods, firePropertyChange, firePropertyChange, firePropertyChange, firePropertyChange, firePropertyChange, getBackground, getBounds, getColorModel, getComponentListeners, getComponentOrientation, getCursor, getDropTarget, getFocusCycleRootAncestor, getFocusListeners, getFocusTraversalKeysEnabled, getFont, getForeground, getGraphicsConfiguration, getHierarchyBoundsListeners, getHierarchyListeners, getIgnoreRepaint, getInputContext, getInputMethodListeners, getInputMethodRequests, getKeyListeners, getLocale, getLocation, getLocationOnScreen, getMouseListeners, getMouseMotionListeners, getMousePosition, getMouseWheelListeners, getName, getParent, getPeer, getPropertyChangeListeners, getPropertyChangeListeners, getSize, getToolkit, getTreeLock, gotFocus, handleEvent, hasFocus, hide, imageUpdate, inside, isBackgroundSet, isCursorSet, isDisplayable, isEnabled, isFocusable, isFocusOwner, isFocusTraversable, isFontSet, isForegroundSet, isLightweight, isMaximumSizeSet, isMinimumSizeSet, isPreferredSizeSet, isShowing, isValid, isVisible, keyDown, keyUp, list, list, list, location, lostFocus, mouseDown, mouseDrag, mouseEnter, mouseExit, mouseMove, mouseUp, move, nextFocus, paintAll, postEvent, prepareImage, prepareImage, remove, removeComponentListener, removeFocusListener, removeHierarchyBoundsListener, removeHierarchyListener, removeInputMethodListener, removeKeyListener, removeMouseListener, removeMouseMotionListener, removeMouseWheelListener, removePropertyChangeListener, repaint, repaint, repaint, resize, resize, setBounds, setBounds, setComponentOrientation, setCursor, setDropTarget, setFocusable, setFocusTraversalKeysEnabled, setIgnoreRepaint, setLocale, setLocation, setLocation, setName, setSize, setSize, show, show, size, toString, transferFocus, transferFocusUpCycle`

**Methods inherited from class java.lang.Object**

`equals, getClass, hashCode, notify, notifyAll, wait, wait, wait`

**Constructor Detail**

### Loader

```
public Loader()
```


**Method Detail**

### globalInfo

```
public java.lang.String globalInfo()
```

:   Global info (if it exists) for the wrapped loader

    :   **Returns:**: the global info

---


### setDB

```
public void setDB(boolean flag)
```

---


### setBeanContext

```
public void setBeanContext(java.beans.beancontext.BeanContext bc)
```

:   Set a bean context for this bean

    :   **Specified by:**: `setBeanContext` in interface `java.beans.beancontext.BeanContextChild` **Overrides:**: `setBeanContext` in class `AbstractDataSource`
    :   **Parameters:**: `bc` - a `BeanContext` value

---


### setLoader

```
public void setLoader(Loader loader)
```

:   Set the loader to use

    :   **Parameters:**: `loader` - a `weka.core.converters.Loader` value

---


### getStructure

```
public Instances getStructure(java.lang.String eventName)
```

:   Get the structure of the output encapsulated in the named
    event. If the structure can't be determined in advance of
    seeing input, or this StructureProducer does not generate
    the named event, null should be returned.

    :   **Specified by:**: `getStructure` in interface `StructureProducer`
    :   **Parameters:**: `eventName` - the name of the output event that encapsulates the requested output. **Returns:**: the structure of the output encapsulated in the named event or null if it can't be determined in advance of seeing input or the named event is not generated by this StructureProduce.

---


### getLoader

```
public Loader getLoader()
```

:   Get the loader

    :   **Returns:**: a `weka.core.converters.Loader` value

---


### setWrappedAlgorithm

```
public void setWrappedAlgorithm(java.lang.Object algorithm)
```

:   Set the loader

    :   **Specified by:**: `setWrappedAlgorithm` in interface `WekaWrapper`
    :   **Parameters:**: `algorithm` - a Loader **Throws:**: `java.lang.IllegalArgumentException` - if an error occurs

---


### getWrappedAlgorithm

```
public java.lang.Object getWrappedAlgorithm()
```

:   Get the loader

    :   **Specified by:**: `getWrappedAlgorithm` in interface `WekaWrapper`
    :   **Returns:**: a Loader

---


### startLoading

```
public void startLoading()
```

:   Start loading data

---


### start

```
public void start()
           throws java.lang.Exception
```

:   Start loading

    :   **Specified by:**: `start` in interface `Startable`
    :   **Throws:**: `java.lang.Exception` - if something goes wrong

---


### getStartMessage

```
public java.lang.String getStartMessage()
```

:   Gets a string that describes the start action. The
    KnowledgeFlow uses this in the popup contextual menu
    for the component. The string can be proceeded by
    a '$' character to indicate that the component can't
    be started at present.

    :   **Specified by:**: `getStartMessage` in interface `Startable`
    :   **Returns:**: a string describing the start action.

---


### eventGeneratable

```
public boolean eventGeneratable(java.lang.String eventName)
```

:   Returns true if the named event can be generated at this time

    :   **Specified by:**: `eventGeneratable` in interface `EventConstraints`
    :   **Parameters:**: `eventName` - the event **Returns:**: a `boolean` value

---


### addDataSourceListener

```
public void addDataSourceListener(DataSourceListener dsl)
```

:   Add a listener

    :   **Specified by:**: `addDataSourceListener` in interface `DataSource` **Overrides:**: `addDataSourceListener` in class `AbstractDataSource`
    :   **Parameters:**: `dsl` - a `DataSourceListener` value

---


### removeDataSourceListener

```
public void removeDataSourceListener(DataSourceListener dsl)
```

:   Remove a listener

    :   **Specified by:**: `removeDataSourceListener` in interface `DataSource` **Overrides:**: `removeDataSourceListener` in class `AbstractDataSource`
    :   **Parameters:**: `dsl` - a `DataSourceListener` value

---


### addInstanceListener

```
public void addInstanceListener(InstanceListener dsl)
```

:   Add an instance listener

    :   **Specified by:**: `addInstanceListener` in interface `DataSource` **Overrides:**: `addInstanceListener` in class `AbstractDataSource`
    :   **Parameters:**: `dsl` - a `InstanceListener` value

---


### removeInstanceListener

```
public void removeInstanceListener(InstanceListener dsl)
```

:   Remove an instance listener

    :   **Specified by:**: `removeInstanceListener` in interface `DataSource` **Overrides:**: `removeInstanceListener` in class `AbstractDataSource`
    :   **Parameters:**: `dsl` - a `InstanceListener` value

---


### main

```
public static void main(java.lang.String[] args)
```

---


### setCustomName

```
public void setCustomName(java.lang.String name)
```

:   Set a custom (descriptive) name for this bean

    :   **Specified by:**: `setCustomName` in interface `BeanCommon`
    :   **Parameters:**: `name` - the name to use

---


### getCustomName

```
public java.lang.String getCustomName()
```

:   Get the custom (descriptive) name for this bean (if one has been set)

    :   **Specified by:**: `getCustomName` in interface `BeanCommon`
    :   **Returns:**: the custom name (or the default name)

---


### setLog

```
public void setLog(Logger logger)
```

:   Set a logger

    :   **Specified by:**: `setLog` in interface `BeanCommon`
    :   **Parameters:**: `logger` - a `weka.gui.Logger` value

---


### setEnvironment

```
public void setEnvironment(Environment env)
```

:   Set environment variables to use.

    :   **Specified by:**: `setEnvironment` in interface `EnvironmentHandler`
    :   **Parameters:**: `env` - the environment variables to use

---


### connectionAllowed

```
public boolean connectionAllowed(java.beans.EventSetDescriptor esd)
```

:   Returns true if, at this time,
    the object will accept a connection via the supplied
    EventSetDescriptor. Always returns false for loader.

    :   **Specified by:**: `connectionAllowed` in interface `BeanCommon`
    :   **Parameters:**: `esd` - the EventSetDescriptor **Returns:**: true if the object will accept a connection

---


### connectionAllowed

```
public boolean connectionAllowed(java.lang.String eventName)
```

:   Returns true if, at this time,
    the object will accept a connection via the named event

    :   **Specified by:**: `connectionAllowed` in interface `BeanCommon`
    :   **Parameters:**: `eventName` - the name of the event **Returns:**: true if the object will accept a connection

---


### connectionNotification

```
public void connectionNotification(java.lang.String eventName,
                                   java.lang.Object source)
```

:   Notify this object that it has been registered as a listener with
    a source for receiving events described by the named event
    This object is responsible for recording this fact.

    :   **Specified by:**: `connectionNotification` in interface `BeanCommon`
    :   **Parameters:**: `eventName` - the event: `source` - the source with which this object has been registered as a listener

---


### disconnectionNotification

```
public void disconnectionNotification(java.lang.String eventName,
                                      java.lang.Object source)
```

:   Notify this object that it has been deregistered as a listener with
    a source for named event. This object is responsible
    for recording this fact.

    :   **Specified by:**: `disconnectionNotification` in interface `BeanCommon`
    :   **Parameters:**: `eventName` - the event: `source` - the source with which this object has been registered as a listener

---


### stop

```
public void stop()
```

:   Stop any loading action.

    :   **Specified by:**: `stop` in interface `BeanCommon`

---


### isBusy

```
public boolean isBusy()
```

:   Returns true if. at this time, the bean is busy with some
    (i.e. perhaps a worker thread is performing some calculation).

    :   **Specified by:**: `isBusy` in interface `BeanCommon`
    :   **Returns:**: true if the bean is busy.


---


|  |  |  |
| --- | --- | --- |
| **Overview** \| **Package** \| **Class** \| **Tree** \| **Deprecated** \| **Index** \| **Help** \| **Weka's home** | |  |
| **PREV CLASS**   **NEXT CLASS** | **FRAMES**    **NO FRAMES**     **All Classes** |  |
| SUMMARY: NESTED \| FIELD \| CONSTR \| METHOD | DETAIL: FIELD \| CONSTR \| METHOD |  |


---
